# Unmet Dental Needs Among Mid-to-Older Deaf and Hard of Hearing Women in the U.S.

**DOI:** 10.3389/froh.2022.866537

**Published:** 2022-05-20

**Authors:** Andrew Donald, Sowmya R. Rao, Katja Jacobs, Nthabeleng MacDonald, Poorna Kushalnagar

**Affiliations:** ^1^Private Dental Practice, Gaithersburg, MD, United States; ^2^Department of Global Health, Boston University School of Public Health, University of Massachusetts Medical Center, Boston, MA, United States; ^3^Center for Deaf Health Equity, Gallaudet University, Washington, DC, United States

**Keywords:** deaf, sign language, women, dental needs, hard of hearing, oral care access, accessibility

## Abstract

**Introduction:**

Despite the significant number of deaf and hard of hearing (DHH) people living in the U.S., oral health research on DHH people who use American Sign Language (ASL) is virtually nonexistent. This study aims to investigate dental needs among mid-to-older DHH women and identify social determinants of health that may place them at higher risk for unmet dental health needs as the primary outcome.

**Methods:**

This cross-sectional study uses data drawn from Communication Health domain in the PROMIS-DHH Profile and oral health data from the National Health and Nutrition Examination Survey. Both measures were administered in ASL and English between November 2019 and March 2020. Univariate and bivariate analysis included only complete data, and multivariable logistic regression analyses were conducted on multiply imputed data.

**Results:**

Out of 197 DHH women (41 to 71+ years old) who answered the dental visit question, 48 had unmet dental needs and 149 had met dental needs. Adjusting for sociodemographic variables, disparity in dental needs was observed across education [OR (95% CI): 0.45(0.15, 1.370)] and communication health [0.95 (0.90, 1.01)].

**Discussion:**

Our study is the first to describe DHH mid-to-older women's access to oral health care. DHH women who do not have a college degree may be impacted. Further research is needed to elucidate the particular risk factors, including cultural, to which DHH individuals from marginalized racial groups are susceptible to unmet oral health needs.

**Conclusions:**

Evidence shows that DHH ASL users who have less years of education or are single experience barriers in accessing dental care.

## Introduction

In the United States, about 9% of the population has hearing loss that may be congenital or age-related, and ~500,000 are American Sign Language (ASL) users [1, 2]. Despite the significant number of DHH people living in the U.S., oral health research on deaf and hard of hearing (which will hereon be referred to as “DHH”) people who specifically use ASL is virtually non-existent. No dental or oral health care studies included measures or survey interviews that are fully accessible to DHH people who use ASL.

Disparities in accessing dental care and maintaining good oral health [3, 4], which are informed by socioeconomic factors, can take a toll on an individual's comprehensive wellbeing [5–7]. Considering these disparities, coupled with the understanding that DHH people have historically faced communication and information barriers in healthcare [8], we intend to determine whether oral health disparities among the general population from marginalized groups are also reflected within the DHH community.

In the general oral health literature, disparities in dental health care are observed among adults who self-identify as Persons of Color (POC), have a high school education or lower, and have low income. Studies have found that untreated cavities have a higher prevalence among non-Hispanic Black or Mexican-American adults and adults with less than a high school education, as well as 40% of low-income adults or adults without private health insurance [5, 7]. Specifically, over 40% of low-income and non-Hispanic Black adults among US adults ages 20–62 have untreated tooth decay. Older adults with low income also experience oral health issues at higher rates, with complete tooth loss being two times as common among adults over 75 than adults aged 65–74 as of 2011-2012 [6]. Untreated oral diseases may impact overall physical health and create a cycle in which already disadvantaged individuals continue to be burdened with high financial costs and poor health outcomes.

The inexorable connection between oral health and overall physical health, including chronic diseases (e.g., cancer, depression, and cardiovascular issues), emphasizes the need to encourage good oral hygiene for all [2, 4, 9]. A large survey conducted in Maryland identified health literacy as playing an essential role in quality of dental care and primary prevention of dental issues and diseases [9]. Research has also identified financial and socioeconomic factors as significant barriers to dental care, oral health literacy, and consequently, good oral health [5, 6]. For example, non-elderly adults were more likely to face financial barriers in all types of care, including dental, than children under 21 years of age by Medicare and elderly adults who have the optional benefit of dental care coverage with Medicaid [10].

As aforementioned above, research clearly supports the finding that barriers to health communication and knowledge are associated with poor health outcomes among DHH people from underserved groups. However, research on oral health in the DHH community that uses ASL is virtually non-existent. This study is the first to date to gather dental needs data from mid-to-older DHH women through a fully accessible survey interview method. Results will be used to identify subgroups who are at disparity for dental needs and also improve oral health outcomes among DHH people who use ASL.

## Materials and Methods

### Measures

This study uses data drawn from the Communication Health Scale in the PROMIS-Deaf Profile and oral health questions in the National Health and Nutrition Examination Survey (NHANES). Below are the communication and oral health items that were asked and used for the purpose of this study.

PROMIS-Deaf Profile: Communication Health [11] (Responses: *Always, Often, Sometimes, Rarely, Never*).

- *I feel my life is good even though I am deaf or hard of hearing*.
- *I am satisfied with the way I communicate with deaf/hh people*.
- *In general, I feel people accept me as a person who is deaf or hard of hearing*.
- *People who are close to me accept me as a person who is deaf or hard of hearing*.
- *I am able to easily get the information I need to make decisions*.
- *People closest to me share the same communication and language*.
- *People close to me make an effort to communicate with me because I am deaf or hard of hearing*.
- *I have friends who are like me and can understand what it is like to be deaf or hard of hearing*.

National Health and Nutrition Examination Survey (NHANES) [12].

- *About how long has it been since {you/SP} last visited a dentist? Include all types of dentists, such as, orthodontists, oral surgeons, and all other dental specialists, as well as dental hygienists*.

### Procedure

Following the institution's human subjects review board's approval, DHH women were recruited through purposive sampling between November 2019 and March 2020. After they filled out an appointment form, a project coordinator contacted them to schedule an interview consisting of two parts: (1) filling out demographics and PROMIS-Deaf Communication Health [11] online survey that is fully accessible in ASL and English, and (2) completing a face-to-face interview with a DHH female research staff fluent in ASL. The face-to-face interview could be completed either in person or through a videoconferencing platform. The total time spent on completing the demographics and NHANES oral health interview was 1 h or less, and each person was given a $25 gift card for their participation.

### Quantitative Analysis Plan

All respondents included in this analysis identified with being female. Sociodemographic variables included age in years, education, race/ethnicity, marital status, health status, and language preference. A new variable was created based on the total number of household members and the total household pre-tax income as a surrogate measure of household poverty. Another measured characteristic included the PROMIS Communication Health T-Score. For a robust sample size to increase the power of racial/ethnic group analysis, African-American/Black group (*n* = 5), Asian/Other (*n* = 7) and Latinx (*n* = 11) were combined into a Persons of Color group. The Persons of Color group (*n* = 23) was then compared to the white group (*n* = 25). Univariate and bivariate analysis included only complete data, and multivariable logistic regression analyses were conducted on multiply imputed data.

Summary statistics [proportions, means and standard deviations (SD)] of sociodemographic and health sample characteristics were obtained for the full female sample, with met and unmet dental health needs. A bivariate analysis tested the association of characteristics and PROMIS Communication Health T-Scores with met and unmet dental health needs in the previous 12 months using a Fisher's Exact Test (categorical variables) or *T*-Test (continuous variables).

Nine (4%), 1(0.5%), 26(13%), 35 (17%) and 49 (24%) subjects had missing data for outcome, education, income, health status and PROMIS Communication Health Score. We created 20 multiply-imputed datasets assuming data was Missing at Random (i.e., missingness depends only on the observed data). Odds Ratios (OR) and 95% Confidence Intervals (CI) were obtained from multivariable logistic regression models fit to each of these datasets. The models evaluated the association of having unmet dental health needs with Age in years (41–50, 51–60, 61–70, ≥71), Race/Ethnicity {white (Ref), Education [HS degree (Ref), Some college, College graduate], Marital Status [Divorced/Widowed/Separated/Never Married (Ref), Married/Living with Partner], and Health Status [Poor/Fair, Good, Very Good/Excellent (Ref)]}. Results were combined from these 20 datasets using the methods described in Rubin [13]. The *p*-values from the 20 regressions were combined as described in Li et al. ([14], Significance levels from repeated *p*-values with multiply-imputed data).

Further analysis was limited to data from DHH mid-to-older women who self-reported having unmet dental health needs. All analyses were conducted in SAS 9.4 (SAS Institute Inc., Cary, NC) and a two-sided *p*-value < 0.05 was considered statistically significant.

## Results

As shown in Table 1, 206 DHH mid-to-older women (48% with < =2 total household members and < =$49,999 total household pre-tax income; 47% married or living with a partner) participated in an interview about their oral health. Majority of the sample self-identified as white (61%). About 39% of the sample had a high school education or lower while 18% completed some college and 43% self-reported having a college degree that could include an associate degree or higher. When asked to rate their health, 44% of the sample self-rated as very good or excellent.

**Table 1:** Distribution of characteristics of 195 women-overall and by unmet dental needs status.

**Variable**	**Overall (*N =* 206)**	**Unmet dental needs**		*p*-value^*^
		**Yes (*N =* 48)**	**No (*N =* 149)**	
	N^**^ (Col%)	N^**^(Row%)		
**Age in years**				<0.01
41–50	32 (15.5)	11 (36.7)	19 (63.3)	
51–60	61 (29.6)	18 (31.6)	39 (68.4)	
61–70	49 (23.8)	13 (27.1)	35 (72.9)	
71+	64 (31.1)	6 (9.7)	56 (90.3)	
**Race/Ethnicity**				0.15
White	125 (60.7)	25 (20.5)	97 (79.5)	
African American/Black	21 (10.2)	5 (26.3)	14 (73.7)	
Asian/Other	32 (15.5)	7 (23.3)	23 (76.7)	
Latinx	28 (13.6)	11 (42.3)	15 (57.7)	
**Education**				<0.01
HS degree	79 (38.5)	27 (36.5)	47 (63.5)	
Some college	37 (18.0)	9 (24.3)	28 (75.7)	
College graduate	89 (43.4)	12 (14.1)	73 (85.9)	
**Total number in household and Total household pre-tax income**				0.61
≤2, ≤49,999	86 (47.8)	23 (27.4)	61 (72.6)	
≤2, ≥50,000	39 (21.7)	6 (16.2)	31 (83.8)	
≥3, ≥50,000	35 (19.4)	8 (24.2)	25 (75.8)	
≥3, ≤49,999	20 (11.1)	5 (27.8)	13 (72.2)	
**Marital status**				0.03
Married/living with partner	96 (46.6)	21 (22.3)	73 (77.7)	
Divorced/separated	51 (24.8)	18 (38.3)	29 (61.7)	
Widowed/never married	59 (28.6)	9 (16.1)	47 (83.9)	
**Health status**				0.02
Poor/Fair	23 (13.5)	11 (50.0)	11 (50.0)	
Good	73 (42.7)	16 (23.2)	53 (76.8)	
Very good/excellent	75 (43.9)	14 (19.4)	58 (80.6)	
**N; Mean (standard deviation)**				
PROMIS communication health T-Score	157; 54.9 (8.9)	38; 50.3 (10.9)	112; 56.6 (7.7)	<0.01

* *Based on Fishers Exact Test or T-Test*.

** *Might not add up to the total due to missing data*.

When asked specifically about visiting a dentist, 197 answered this question and were therefore included in subsamples of unmet dental needs (*n* = 48) and met dental needs (*n* = 149). Between these two subsamples, disparity was observed across multiple characteristics. Nine DHH women did not indicate whether their needs were met or not. Table 1 shows that unmet needs were higher among the younger DHH women with lower education and lower income than their counterparts (older, higher education and higher income). Thirty-one percent (*n* = 11) of Persons of Color had unmet dental needs compared with 21% (*n* = 25) of White and 23% (*n* = 7) of Asian/Other DHH women.

The proportion of unmet dental needs was higher for divorced/separated women compared to other groups. For example, 38% (*n* = 18) of divorced/separated women had unmet dental needs while only 22% (*n* = 21) of women who were married or living with partners 16% (*n* = 9) of those widowed/never married had unmet needs. The unmet dental needs were proportionally higher for DHH women who self-reported their health as being poor or fair (50%) compared to DHH women whose reported health were very good or excellent (19%). The average PROMIS communication health score was lower among those with unmet needs (mean = 50.3; SD = 10.9) than those without unmet needs (56.6; 7.7) (*p* < 0.01).

After adjusting for other variables in Table 2, results from multivariable logistic regression model indicate that education was statistically significant (*p* = 0.02) for unmet dental needs. Compared with DHH women with a high school education or lower, those with some college had 55% reduced odds [OR (95% CI): 0.45 (0.15, 1.37)] and those with a college education had 76% reduced odds [OR (95% CI): 0.24 (0.09, 0.64)] of having unmet oral needs. In other words, DHH women who did not have any years of college education had higher odds of experiencing unmet dental needs.

**Table 2:** Odds Ratios (OR) and 95% Confidence Interval (CI) obtained from logistic regressions to evaluate the association of characteristics with unmet dental needs (*n* = 48).

**Characteristics**	**OR (95% CI)**	P-Value^*^
***Age in years***		0.02
41–50	*Ref*.	
51–60	0.67 (0.20,2.20)	
61–70	0.54 (0.14,2.16)	
71+	0.12 (0.03,0.59)	
***Race/Ethnicity***		0.42
White	*Ref*.	
Persons of color	1.47 (0.58,3.77)	
***Education***		0.02
HS degree	*Ref*.	
Some college	0.45 (0.15,1.37)	
College graduate	0.24 (0.09,0.64)	
***#HH/Income***		0.66
< =2, < =$49,999	*Ref*.	
< =2, >=$50,000	0.79 (0.24,2.56)	
>=3, >=$50,000	0.63 (0.17,2.28)	
>=3, < =$49,999	0.38 (0.08,1.89)	
***Marital status***		0.02
Widowed/Never married	*Ref*.	
Married/Living with partner	2.59 (0.92,7.29)	
Divorced/Separated	4.82 (1.62,14.32)	
***Health status***		
Poor/Fair	*Ref*.	0.38
Good	0.45 (0.14,1.47)	
Very good/Excellent	0.60 (0.16,2.19)	
***PROMIS comm health T-score***	0.95 (0.90,1.01)	0.10

* *Odds Ratios (OR) and 95% Confidence Intervals (CI) were obtained from multivariable logistic regression models fit to each of 20 multiply-imputed datasets assuming data was Missing at Random (i.e. missingness depends only on the observed data). The p-values from the 20 regressions were combined as described in Li et. al. [14]*.

Although not statistically significant, the odds of unmet needs were relatively higher for those who were Persons of Color compared to white [1.47 (0.58, 3.77)], single compared to those who are married or living with partner [2.59 (0.92, 7.29)]; and divorced or separated [4.82 (1.62, 14.32)] compared with those widowed or never married. The odds decreased with increasing age from 0.67 (0.20, 2.20) for 51–60 years to 0.12 (0.03, 0.59) for 71+ years, and were lower for those who had 3 or more number of household members and < =$49,999 income [0.38 (0.08, 1.89] than those with < =2 members and < =$49,999 total household income, and those with very good/excellent health [0.60 (0.16, 2.19)] compared with those reporting poor/fair health. The odds of unmet needs also decreased per unit of increase in PROMIS Communication Health T-Score [0.95 (0.90, 1.01)].

## Discussion

Our study is the first to describe DHH mid-to-older women's access to oral health care. All DHH women in our sample used ASL. Within this sample, DHH women who reported low-perceived communication health (e.g., not being accepted as a DHH person by others; difficulty with letting others know about their communication needs) were more likely to have unmet dental needs. This is consistent with a national patient-reported outcomes study that reported lower Global Health status (worse overall health) among DHH adults with worse communication health scores [11]. The Kushalnagar et al. study also found that worse communication health scores were associated with lower levels of education and self-identifying as belonging to a marginalized group.

DHH women with financial struggles may not have easy access to oral health care and those who do not have a college degree may be impacted even more. A review of the literature identifies financial issues as a significant barrier to dental care and good oral health [5, 6]. Our study's finding of an inverse relationship between age and unmet needs is consistent with a study that reported younger adults being at higher risk for financial barriers to dental care [10]. Our results highlight how the dental care system is characterized by inequities that place younger or uneducated DHH women at higher risk for adverse dental health outcomes. Efforts in not only making communication accessible but also taking the time to educate them can have a positive impact in ensuring that their dental needs are met.

A well-designed study conducted by Schneidewind et al., [15] simulated DHH patients who needed new appointments in health and dental offices. This study found that, compared to medical offices, dental offices were far more likely to deny appointment requests from new DHH patients because they needed an interpreter to communicate with the dentist, despite the existence of the federal law that requires effective communication in medical and dental offices. The authors speculated that the higher rejection rate was because dental clinics were less likely to be affiliated with a health care system that may be more equipped to provide accessibility and accommodation. Regardless, communication and attitudinal barriers toward DHH people must be removed to ensure that the DHH individual receives equitable access to dental care. Sign language interpreters are readily available and accessible to accommodate DHH women who communicate best in ASL. For DHH women who prefer English-based communication, speech-to-text transcription or writing on a whiteboard can help clarify potential misunderstandings between the DHH patient and dentist.

Dentists should not stop at communication-related accessibility; they must also take appropriate actions to mitigate social determinants that contribute to poor dental health outcomes. Literature shows that if an individual has adverse childhood experiences in their early years and does not have a college education, they are more likely to experience unmet dental care compared to individuals who have had access to education and health [16]. For our DHH sample, we speculate that the likelihood of dental care being unmet is much higher when a DHH individual self-identifies as belonging to a marginalized racial or ethnic group, does not have a college education, and uses ASL.

While it is not possible to conduct intersectional analysis on multiple intersecting variables (e.g., age, race/ethnicity), we did find an increased odds of unmet needs among mid-to-older DHH women of color after adjusting for correlates. This finding is consistent with research that reported Mexican Americans as less likely to receive preventative dental care and being more likely to experience adverse oral health outcomes, such as periodontitis, in comparison to other ethnic groups in the U.S. [17, 18]. Research has also shown that individuals who are black and U.S. natives or those who are Latinx and do not speak English in the U.S. disproportionately experience barriers to dental care, driven by linguistic and cultural communication barriers, lack of oral health knowledge, and financial difficulties, which may explain why DHH women of color who use ASL reported greater odds of unmet oral health needs in comparison to DHH women who self-identify as white [19, 20]. The intersectionality of multiple marginalized identities is likely to exacerbate the barriers to receiving adequate oral health care among the DHH community. Further research is needed to elucidate the particular risk factors, including cultural, to which DHH individuals from marginalized racial/ethnic groups are susceptible to unmet oral health needs.

Suggested actions toward reducing oral health disparities for DHH patients with less years of education or low oral health literacy include allowing as many questions as possible to be asked and taking time to educate DHH patients. Some DDH patients may carry a belief that dental visits should be reserved for pain-related reasons. Dental schools typically provide cultural humility training as part of their dental curriculum, and dentists are responsible to educate the patients about the importance of dental health care while respecting patients as individuals with a wide range of experiences and beliefs. Dental schools are also often affiliated with a health care system that is equipped to provide interpreting services; dental teams should work together to ensure that interpreting services are set up to accommodate DHH dental patients. Dentists who encounter these DHH patients have the responsibility to accept them as patients and provide appropriate accommodation, including in-person interpreting and video remote interpreting if preferred by the DHH patient (see [21], for a review).

There is much that needs to be done to move toward dental equity for all. More people face financial barriers to accessing dental care more than any other type of care, regardless of socioeconomic status [10]. For DHH individuals, receiving dental care may be made even more difficult when encountering communication barriers in healthcare settings [8]. The American Dental Association established guidelines for how to work with DHH patients and recommends securing an interpreter to accommodate dental patients who require one to communicate [22]. Yet, many dentists in small private offices may not encounter DHH patients often in their care and may be less familiar with resources as to where to secure qualified interpreters. As such, accessibility resources (e.g., interpreting services or communication services for the deaf) need to be made widely available for dentists who may not be familiar with the procedure associated with making interpreting service requests.

### Limitations

Study limitations include a majority of white-identified, fully women-identified mid-to-older DHH respondents, self-selected respondents. Although the full sample is large (*N* = 204) given the nature of our low incidence and hard-to-reach population of ASL-using DHH women, the number of DHH mid-to-older women with unmet dental needs is small (*n* = 48), which limited our ability to conduct intersectional analyses. An in-depth qualitative study is recommended to identify recurrent issues that emerge in subsamples of DHH women with intersecting identities.

### Future Directions and Recommendations

There is evidence that DHH ASL users experience barriers in accessing not only health care but also dental care. It is not surprising that a subset of DHH ASL users with lower education are at higher risk for unmet dental care. Communication breakdowns between the patient and provider risk misdiagnosis and misconceptions regarding a DHH patient's health if accessible communication services are not readily available [15]. Given the link between communication health and unmet dental needs in our study, dental health professionals and advocates must work together to address the systemic gaps in dental health care and identify strategies to prevent communication barriers that may be associated with missed diagnoses and delayed oral treatments.

## Data Availability Statement

The datasets presented in this article are not readily available because this is a marginalized subgroup of mid-to-older DHH women in a small community that uses ASL. The dataset may include identifiable information. Requests to access the datasets should be directed to poorna.kushalnagar@gallaudet.edu.

## Ethics Statement

The studies involving human participants were reviewed and approved by Gallaudet University IRB Committee. The patients/participants provided their written informed consent to participate in this study.

## Author Contributions

AD contributed to conception of the oral health study. PK designed the study, oversaw data collection, and drafted the methods section. SR performed the statistical analysis and drafted the results section. AD and PK interpreted the results. KJ and NM assisted with the literature review. All authors contributed to manuscript, read, and approved the submitted version.

## Funding

This work was supported by National Institute on Deafness and Other Communication Disorders (NIDCD) of the National Institutes of Health (R01DC014463-01A1 and R01DC014463-05S1 to PK).

## Conflict of Interest

The authors declare that the research was conducted in the absence of any commercial or financial relationships that could be construed as a potential conflict of interest.

## Publisher's Note

All claims expressed in this article are solely those of the authors and do not necessarily represent those of their affiliated organizations, or those of the publisher, the editors and the reviewers. Any product that may be evaluated in this article, or claim that may be made by its manufacturer, is not guaranteed or endorsed by the publisher.
